# Pyriform Sinus Tract Injury After Traumatic Intubation With Resulting Tension Pneumothorax

**DOI:** 10.31486/toj.18.0155

**Published:** 2020

**Authors:** John N. Cefalu, Danielle C. Williams, Tejas V. Joshi, Alan David Kaye

**Affiliations:** ^1^ Department of Anesthesiology, Louisiana State University Health Sciences Center, New Orleans, LA; ^2^ Department of Oral and Maxillofacial Surgery, Louisiana State University Health Sciences Center, New Orleans, LA; ^3^ Department of Medicine, Louisiana State University Health Sciences Center, New Orleans, LA

**Keywords:** *Intubation–intratracheal*, *pharynx*, *pneumothorax*, *pyriform sinus*

## Abstract

**Background:** Tension pneumothorax is a serious, potentially life-threatening condition with numerous etiologies. Hypopharyngeal injury, a possible complication of endotracheal intubation, can lead to tension pneumothorax. We describe a hypopharyngeal injury that occurred during endotracheal intubation that resulted in tension pneumothorax.

**Case Report:** A 30-year-old female underwent emergent chest tube placement after sustaining an intraoperative tension pneumothorax caused by pyriform sinus tract injury during traumatic intubation for an elective fistulectomy, debridement of a previous fracture site, and removal and replacement of hardware 4 months from the time of the initial injury. A timely chest x-ray aided in the discovery of the pneumothorax. Postoperatively, the patient's tension pneumothorax resolved, her chest tube was removed, and she was extubated during her stay in the trauma intensive care unit. The patient was discharged without any other major complications.

**Conclusion:** Tension pneumothorax is a rare but reported complication that can occur during intubation and intraoperatively. Early recognition of hypopharyngeal perforation is crucial to successful management. Anticipation of a difficult airway can suggest the use of alternative methods of intubation that may reduce the risk of hypopharyngeal perforation.

## INTRODUCTION

Endotracheal intubation is considered a safe and reliable method for securing an airway in the operating room, intensive care unit, and the emergency department when performed by an experienced provider. However, intubation can be harmful if it results in trauma. While complications of endotracheal intubation are uncommon, hypopharyngeal injury is one possible complication and can be associated with devastating complications, including tension pneumothorax, mediastinitis, and pneumomediastinum.^1^ The pharynx, posterior to the cricopharyngeal muscle, is the most common site of hypopharyngeal injury, followed by the pyriform sinus.^2^ A perforating injury to either side of the laryngeal orifice can result in sinus tract formation and lead to fatal complications.^1^ Early findings after hypopharyngeal injury include subcutaneous and mediastinal emphysema.

We present a case of preoperative tension pneumothorax development secondary to pyriform sinus tract formation during endotracheal intubation.

## CASE REPORT

A 30-year-old female with a medical history of hypertension and smoking sustained a left mandibular angle fracture after a fall from a ladder. The patient underwent open reduction and internal fixation of her left mandibular angle through an intraoral approach without complications. The patient developed an infection at the site of the hardware and nonunion of the fracture site as a result of noncompliance—lack of smoking cessation and poor oral hygiene—with postoperative instructions. For treatment of the nonunion and infection, the patient returned to the operating room (OR) approximately 1 month after the initial surgery for debridement and removal and replacement of the hardware. Two months from the time of injury, the patient sustained a fist-to-face assault and developed an abscess at the site of the hardware. Incision and drainage of the abscess were performed at an outside hospital. However, the patient developed an extraoral fistula at the site of the infected hardware. She was scheduled for an elective fistulectomy, debridement of the previous fracture site, and removal and replacement of hardware 4 months from the time of her initial injury.

In the OR, all standard American Society of Anesthesiologists monitors were placed. The patient underwent preoxygenation for 8 minutes prior to intubation, and induction of general anesthesia was facilitated with 80 mg lidocaine, 160 mg propofol, 150 mcg fentanyl, 120 mg succinylcholine, and 10 mg rocuronium. Nasoendotracheal intubation was performed with a 7.5 mm nasal right angle endotracheal (RAE) tube, and a MacIntosh 3.5 laryngoscope was placed with direct laryngoscopy; however, 2 minutes after intubation during confirmation of tube placement, the patient was found to have a leak related to cuff rupture. An Eschmann-guided exchange was performed with a 7.0 mm nasal RAE tube and a McGrath video laryngoscope because a Cook airway exchange catheter was not available. Placement was confirmed with bilateral breath sounds, symmetric chest rise, and capnography; however, the patient had diminished breath sounds throughout auscultation with expiratory wheezes bilaterally. Approximately 10 minutes following confirmation of endotracheal tube placement, the patient's peak inspiratory pressures increased from 23 cm H_2_0 to 64 cm H_2_0, resulting in difficult ventilation. Numerous measures were taken, including suctioning the nasal RAE tube and administering 0.5 mg terbutaline subcutaneously and 0.5 mL of 2.25% nebulized racemic epinephrine; however, no improvement in ventilation and peak pressures was noted. Six minutes after the elevation in peak pressures, the 7.0 mm nasal RAE tube was removed, and bag-mask ventilation was initiated. Peak pressures elevation and ongoing difficulty with ventilation continued. The patient's oxygen saturation declined from 98% to 54%, and her heart rate decreased from 104 to 64 bpm, but no significant decline in blood pressure was noted. The patient was reintubated using a McGrath video laryngoscope and a 7.0 mm oral endotracheal tube, and ventilation was initiated via Ambu bag and supplemental oxygen. The patient's oxygen saturation increased to 96% and her heart rate to 92 bpm.

**Figure 1.**
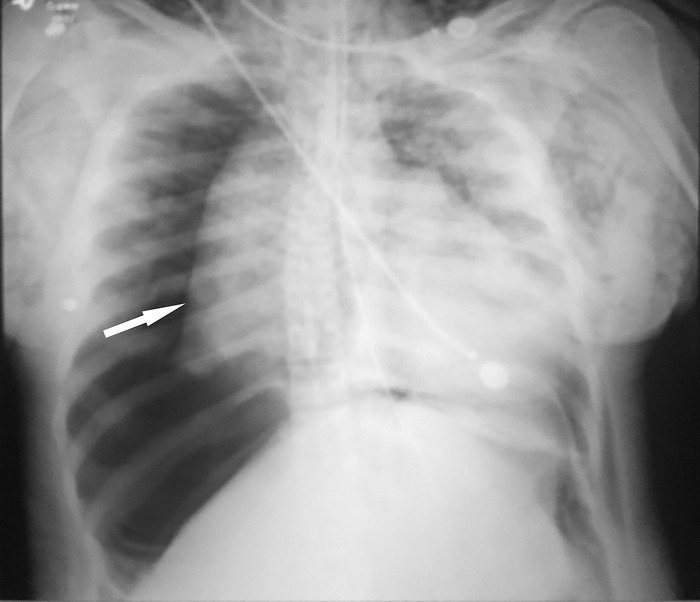
**Intraoperative chest x-ray shows bilateral subcutaneous air, pneumomediastinum, and a large right-sided tension pneumothorax (white arrow).**

She began to develop significant left-sided facial and bilateral neck and chest edema with crepitus, indicating subcutaneous air. Decreased right breath sounds were noted with auscultation. Intraoperative chest x-ray revealed significant bilateral subcutaneous air, pneumomediastinum, and a large right-sided tension pneumothorax (Figure 1). An attempt at needle decompression failed because of large chest wall thickness. General surgery emergently placed a 32-French chest tube on the right side without complication, resulting in an almost immediate improvement in ventilation and tidal volumes and resolution of the tension pneumothorax. The fistulectomy, debridement, and removal of hardware procedures were aborted.

**Figure 2.**
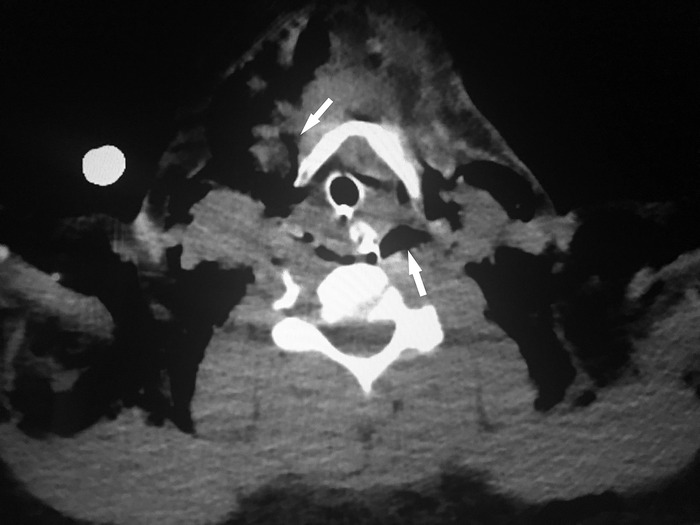
**Computed tomography of the neck shows subcutaneous air in the anterior and posterior compartments of the neck (white arrows).**

**Figure 3.**
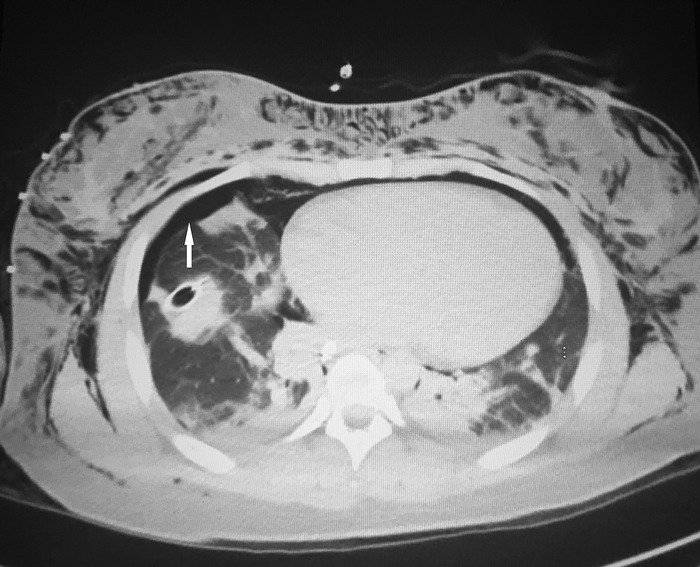
**Postoperative computed tomography of the chest shows subcutaneous air and right-sided pneumothorax (white arrow).**

The patient was transferred to the trauma intensive care unit (TICU) where she remained intubated and sedated. Bronchoscopy performed intraoperatively in the TICU following chest tube placement revealed no obvious injuries. Postoperative computed tomography (CT) maxillofacial scan without contrast revealed extensive air within the right parotid, masticator, retropharyngeal, and bilateral carotid spaces, along with right preseptal periorbital, bilateral neck, and anterior chest wall soft tissues. CT of the neck without contrast demonstrated a large amount of soft tissue emphysema within the anterior and posterior neck, superior mediastinum, and chest wall compartments, and CT of the chest without contrast demonstrated persistent right-sided pneumothorax (Figures 2 and 3).

**Figure 4.**
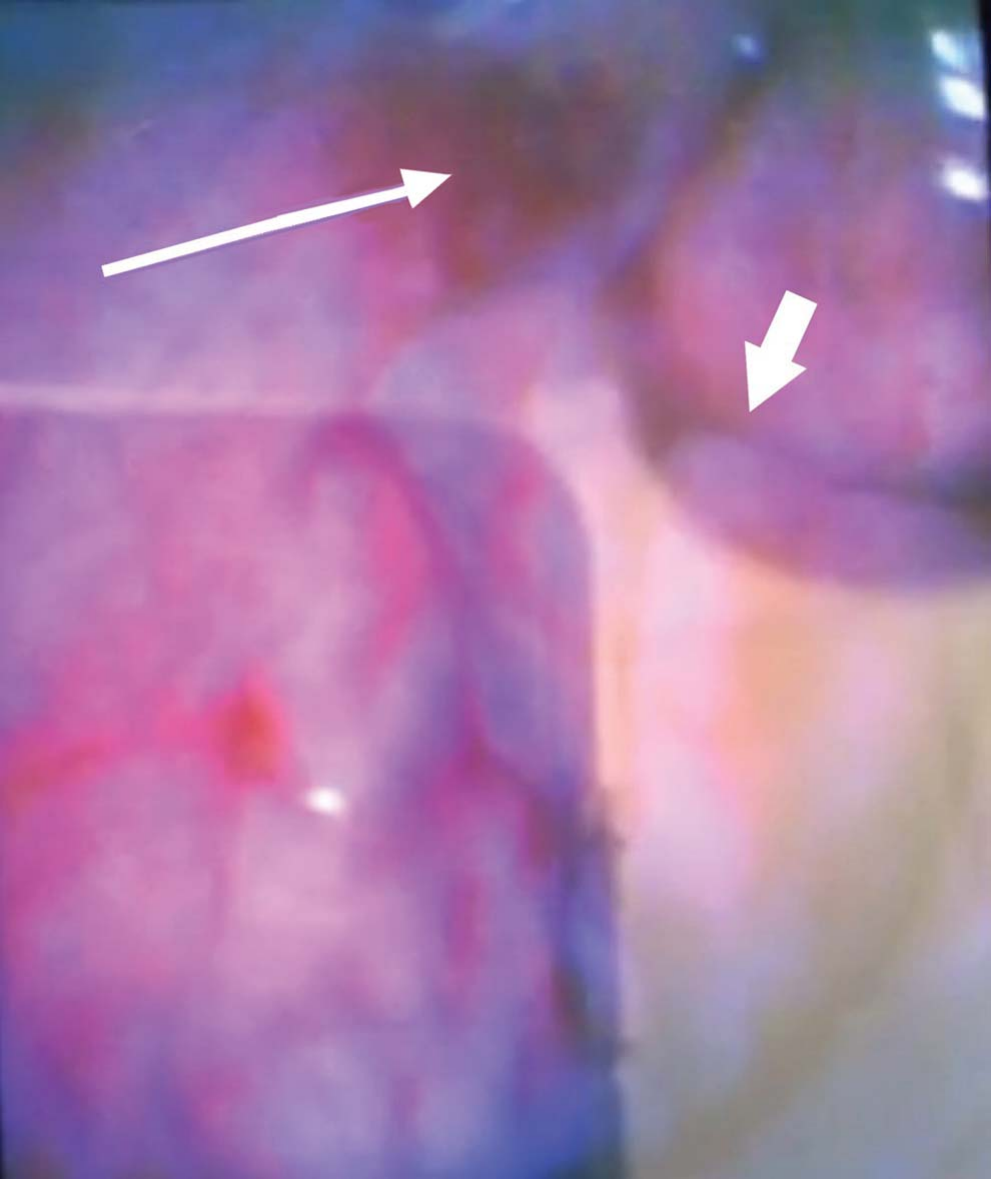
**Image from the McGrath video laryngoscopy demonstrates hypopharyngeal pyriform sinus injury (long, thin arrow) and epiglottis (short, thick arrow).**

The following day, flexible fiberoptic laryngoscopy performed by the TICU service revealed a tissue flap in the posterior hypopharynx with overlying blood clot. After removal of the clot, a tissue defect consistent with a right pyriform sinus tract laceration injury was identified (Figure 4). Otolaryngology confirmed the pyriform sinus injury. Enteral feeding through an orogastric tube was initiated, and intravenous (IV) ampicillin-sulbactam was administered for aerodigestive organism coverage.

The patient remained intubated and sedated until day 5 when she was extubated over an Eschmann catheter by the anesthesia service. The following day, the patient's chest tube was removed, and she was transferred to the floor for monitoring of her hypopharyngeal injury. She remained an inpatient for 2 days and then requested discharge from the hospital. The patient was advised to remain hospitalized until the otolaryngology service could perform a barium esophagram with laryngoscopy to reevaluate the pyriform sinus laceration, but the patient left the hospital against medical advice on postoperative day 8 after the risks had been discussed with her. The patient completed 8 of the recommended 10 days of IV ampicillin-sulbactam and 8 days of enteral feeding.

The patient was successfully contacted 1 week after leaving the hospital and agreed to return for follow-up evaluation and another attempt to perform the procedure. One month later, the patient underwent fistulectomy, debridement of the previous fracture site, and removal and replacement of hardware after nasoendotracheal intubation without complication using a C-MAC video laryngoscope. Preoperative evaluation with awake nasolaryngoscopy performed by the otolaryngology service revealed a well-healed pyriform sinus. Surgery was completed without incident, and the patient was extubated postoperatively without incident.

The patient was seen postoperatively 3 times in 1 month for outpatient follow-up. She continued to smoke cigarettes daily and did not comply with the recommended no-chew diet. At her final visit 1 month postoperatively, she had developed a superficial intraoral surgical site dehiscence. She did not return for additional follow-up visits.

## DISCUSSION

The common requirement for nasoendotracheal intubation during maxillofacial surgical procedures can be challenging for the anesthesiologist, especially in patients with difficult airways. Multiple types of injury have been reported during endotracheal intubation or procedures utilizing scopes in the aerodigestive tract, including transesophageal echocardiograms and esophagogastroduodenoscopy.^3^

Predictive measures of a difficult airway, such as a Mallampati score greater than III, a class III upper lip bite test or the inability to bite the upper lip with the mandible, head and neck range of motion <90°, an incisor gap <4 cm, the presence of prominent central incisors, a thyromental distance <6 cm, and a total airway score (TAS) >6, can help healthcare professionals predict a difficult intubation.^4^ The TAS utilizes all 6 of the aforementioned predictors of difficult intubation.

If a difficult airway is anticipated, the anesthesiologist can consider alternatives to the traditional methods of intubation with Macintosh or Miller laryngoscopes, such as video laryngoscope or fiberoptic bronchoscope. Video laryngoscopy success rates are superior to traditional laryngoscopes in first intubation for patients predicted preoperatively to have difficult intubation,^5^ possibly because the video laryngoscope improves the ability to visualize the glottic opening.^6^ Tabrizi et al demonstrated that despite an increased intubation time, video laryngoscopy significantly increased the first-attempt success rate of nasoendotracheal intubation in patients with bilateral mandible fractures.^7^

Our patient had a Mallampati score of IV but a thyromental distance >6.5 cm, normal neck range of motion, incisor gap >4 cm, upper lip bite test class I, and lack of prominent incisors. Because the patient had 1 predictor of difficult intubation, the initial use of video laryngoscopy may have reduced the number of attempts at intubation and thus prevented hypopharyngeal injury.

Early recognition of perforation is crucial to proper management, as shown in our case. The timely chest x-ray revealed the pneumothorax that was addressed immediately. The most frequently perforated sites in the hypopharynx are the pharynx, posterior to the cricopharyngeal muscle, followed by the pyriform sinus.^2^ In a case similar to ours, laceration of the right posterior hypopharynx during multiple attempts at intubation led to extensive pneumomediastinum and right-sided cervical subcutaneous emphysema.^1^ Other case reports of iatrogenic pyriform sinus lacerations demonstrate that they can cause complications such as mediastinal perforation and massive subcutaneous emphysema.^8,9^

Perforation of the aerodigestive tract can have serious sequelae, such as mediastinitis or infection that can lead to an abscess or sepsis.^1^ Treatment for perforation ranges from conservative management to surgical repair. The choice of treatment varies, depending on the severity and location of injury. If feasible, the conservative approach entails initiating broad-spectrum IV antibiotic therapy, endotracheal intubation, and nasogastric tube insertion for enteral feeding.^10^ Our patient successfully recovered with 5 days of oroendotracheal intubation and enteral feeding for 8 days. Ideally, care for these patients is multidisciplinary, with involvement of otolaryngologists, speech pathologists, intensive care specialists, and anesthesia providers.

Tension pneumothorax is a rare but reported complication that can occur during intubation and intraoperatively.^11^ Airway exchange catheters such as the Eschmann have been associated with bronchial injury and tension pneumothorax.^12,13^ Therefore, judicious and gentle use of these catheters may reduce the likelihood of this complication.

## CONCLUSION

This complex case highlights the importance of early recognition of hypopharyngeal perforation and tension pneumothorax, as well as a timely chest x-ray and chest tube placement. Although preventing all injuries is not possible, certain measures can increase the possibility of a nontraumatic and successful first attempt at endotracheal intubation, including anticipating a difficult airway and using alternative methods of intubation such as video laryngoscopy instead of traditional Macintosh and Miller laryngoscopes. Perforation of the aerodigestive tract should first be treated conservatively with IV broad-spectrum antibiotic therapy, endotracheal intubation, and insertion of nasogastric tube for enteral feeding with a multidisciplinary team involving otolaryngologists, speech pathologists, intensive care specialists, and anesthesia providers.

## Notes

This article meets the Accreditation Council for Graduate Medical Education and the American Board of Medical Specialties Maintenance of Certification competencies for Patient Care and Medical Knowledge.
